# The Role of Peroxisome Proliferator-Activated Receptors in PGF_2α_-Induced Luteolysis in the Bovine Corpus Luteum

**DOI:** 10.3390/ani12121542

**Published:** 2022-06-14

**Authors:** Barbara Maria Socha, Piotr Łada, Agnieszka Walentyna Jończyk, Anna Justyna Korzekwa, Dariusz Jan Skarżyński

**Affiliations:** 1 Department of Reproductive Immunology and Pathology, Institute of Animal Reproduction and Food Research, Polish Academy of Sciences, Tuwima 10, 10-748 Olsztyn, Poland; a.jonczyk@pan.olsztyn.pl (A.W.J.); d.skarzynski@pan.olsztyn.pl (D.J.S.); 2 Veterinary Clinic 3VET, Ludowa 78/3, 18-200 Wysokie Mazowieckie, Poland; lada_wet@o2.pl; 3 Department of Biodiversity Protection, Institute of Animal Reproduction and Food Research, Polish Academy of Sciences, Tuwima 10, 10-748 Olsztyn, Poland; a.korzekwa@pan.olsztyn.pl

**Keywords:** peroxisome proliferator-activated receptor, corpus luteum, prostaglandin F_2α_, estrous cycle, luteolysis, cow

## Abstract

**Simple Summary:**

The corpus luteum (CL) is responsible for progesterone (P_4_) secretion. In the absence of pregnancy, luteolysis occurs, which leads to a reduction in P_4_ production, followed by the structural regression of the CL. In cows, prostaglandin F_2α_ (PGF_2α_) is the main luteolytic factor. It is also an endogenous ligand for peroxisome proliferator-activated receptors (PPARs), which are important factors regulating mammalian reproductive function. However, the mechanisms of action of PPAR isoforms, i.e., PPARα, PPARδ and PPARγ, in the luteolytic pathways in cattle are still not fully understood. The aim of this in vitro study was to determine the expression of PPAR isoforms in the bovine CL throughout the estrous cycle, and their involvement in PGF_2α_-induced processes related to luteolysis. The obtained results indicate that the expression of PPARs changes in the bovine CL throughout the estrous cycle; moreover, PGF_2α_ affects its expression. This study provides evidence that PPARγ, among all examined PPAR isoforms, could be involved in the regulation of PGF_2α_-induced luteolysis in cattle, and PPARs may affect CL regression at multiple sites. These results help to widen the knowledge of the mechanisms of luteal regression in the bovine CL.

**Abstract:**

The participation of peroxisome proliferator-activated receptors (PPARs) in ovarian function in cattle is still not fully understood. The aim of this in vitro study was to determine: (i) the immunolocalization, mRNA expression and tissue concentration of PPARα, PPARδ and PPARγ in the bovine corpus luteum (CL) (*n* = 40) throughout the estrous cycle, and (ii) the involvement of PPAR in PGF_2α_-induced processes related to luteolysis. CL (*n* = 9) explants were cultured in the presence of PPAR antagonists (10^−5^ M) in combination with or without PGF_2α_ receptor antagonist (10^−5^ M) and PGF_2α_ (10^−6^ M). The mRNA and protein expression of PPARs was evaluated through qPCR, IHC, and ELISA, respectively. The results showed that PPAR mRNA and protein expression differed according to the luteal stages. PGF_2α_ upregulated *PPARδ* and *PPARγ* mRNA expression in the bovine CL in vitro, whereas PPARγ increased the inhibitory effect of PGF_2α_ by decreasing progesterone secretion and the mRNA expression of hydroxy-delta-5-steroid dehydrogenase, 3 β- and steroid delta-isomerase 1 (*HSD3B1*) in the CL explants; mRNA transcription of tumor necrosis factor α (*TNFα*) and inducible nitric oxide synthase (*iNOS*) was increased. The obtained results indicate that the mRNA and protein expression of PPARs changes in the bovine CL throughout the estrous cycle and under the influence of PGF_2α_. We suggest that isoform γ, among all examined PPARs, could be a factor involved in the regulation of PGF_2α_-induced processes related to luteolysis in the bovine CL. Further studies are needed to understand the role of PPAR in luteal regression in the CL of cattle.

## 1. Introduction

Peroxisome proliferator-activated receptors (PPARs) are a family of nuclear receptors (NRs) that comprise three isoforms: alpha (PPARα; NR1C1), delta (PPARδ; NUC1; NR1C2) and gamma (PPARγ; NR1C1), which are encoded by different genes [1,2]. They are ligand-dependent multifunctional transcription factors that, through the transcriptional regulation of target gene expression, enable the cell to respond to extracellular stimuli [2].

The influence of PPARs on ovarian function is still not fully understood. The most extensively studied PPAR isoform is PPARγ relative to the other two isoforms, and it has been detected in mouse [3], rat [4], pig [5], sheep [6], and human [7] ovaries. Studies on rodents and humans have revealed that PPARγ modulates gametogenesis, ovulation and corpus luteum (CL) formation or regression by participating in the regulation of genes controlling steroidogenesis, angiogenesis and tissue remodeling and inflammatory response [8,9,10,11].

It is known that many factors activate PPARs and have well-established roles in the biology of the ovaries. For example, endogenous factors that have been shown to activate PPARs and also influence ovarian functions are fatty acids and eicosanoids, i.e., prostaglandins (PGs) [12]. Their presence can either stimulate or inhibit receptor functions [12]. An interaction between PPARs and PGs has been suggested in mammary human epithelial cells, as the peroxisome proliferator response element (PPRE) was detected in the prostaglandin-endoperoxide synthase-2 (PTGS2, COX-2) promoter, which is a key enzyme that is responsible for the synthesis of prostaglandin F_2α_ (PGF_2α_) [13]. Additionally, in our previous studies, we observed that under the influence of PGF_2α_, the mRNA expression of PPARγ increased in bovine endometrial stromal cells [14].

In cattle, PGF_2α_ is the main luteolytic factor [15]. It induces luteolysis in vivo approximately between days 15 and 17 of the cycle or when exogenously administered during the mid-luteal phase in cows, through the endocrine effects on CL [15,16,17,18,19,20,21]. The cascade of CL regression consists of (i) functional luteolysis (interruption of steroidogenesis and decreasing P_4_ production) and (ii) structural luteolysis (degradation/demise of CL tissue due to cell death) [22,23]. It is known that many factors, such as tumor necrosis factor α (TNF_α_), interferon γ (IFN_γ_), nitric oxide (NO) and leukotriene C_4_ (LTC_4_), act as intra-luteal mediators of the luteolytic action of PGF_2α_ [24,25,26,27,28,29,30]. However, the direct influence of PGF_2α_ on bovine CL in vitro is still controversial and depends on the methodology of examination, as previously PGF_2α_ stimulated [22,28,31,32,33], inhibited [34], or had no direct effect on P_4_ secretion in cultured steroidogenic luteal cells [35].

Studies suggesting the involvement of PPARs in regulating ovarian functions in cows, with a particular emphasis on the function of CL, are limited. To date, only PPARγ activity has been noted in the bovine large luteal cells [36,37]. Its expression increased after ovulation; however, if fertilization did not occur, the CL regressed, and PPARγ expression decreased [36,37]. There are no data describing the relationship between PPARα, PPARδ and PPARγ expression in the bovine CL during the estrous cycle regarding the luteolytic activity of PGF_2α_ as a PPAR ligand and the potential influence of PPAR isoforms on PGF_2α_-induced processes related to functional luteolysis. Therefore, we hypothesized that in the bovine CL, the expression of PPAR isoforms depends on the phase of the estrous cycle and that their expression is changing under the influence of PGF_2α_, and PPARs could be involved in the modulation of PGF_2α_-induced processes related to luteolysis (in vitro).

The aim of this study was to determine: (i) the immunolocalization, mRNA expression and tissue concentrations of PPARα, PPARδ and PPARγ in the bovine CL throughout the estrous cycle, and (ii) whether PPARs could mediate PGF_2α_ actions during regression of the bovine CL. The possible involvement of PPARα, PPARδ and PPARγ in the luteolytic actions of PGF_2α_ was studied in vitro, and selected factors and mediators of the luteolytic cascade were measured using the mRNA expression of (1) steroidogenic enzymes: steroidogenic acute regulatory protein (StAR), cytochrome P450 family 11 subfamily A member 1 (P450scc), hydroxy-delta-5-steroid dehydrogenase, 3 β- and steroid delta-isomerase 1 (HSD3B1); (2) enzymes responsible for arachidonic acid (AA) metabolism: prostaglandin-endoperoxide synthase-2 (PTGS2) and prostaglandin F_2α_ synthase (PTGFS); (3) selected factors mediating luteolysis: tumor necrosis factor α (TNFα), tumor necrosis factor receptor superfamily member 1A (TNFRSF1A), tumor necrosis factor receptor superfamily member 1B (TNFRSF1B) and inducible nitric oxide synthase (iNOS) [22,23,24,25,26,27,28,29,30].

## 2. Materials and Methods

### 2.1. Animal and Material Collection

Corpora lutea (CL) were collected from the same heifers, which have been previously described [38]. In brief, healthy, normally cycling Holstein/Polish Black and White (75% and 25%, respectively) heifers (aged between 18 and 22 months) were used for the present study. An experienced veterinarian using ultrasound examination (USG) per rectum with a 7.5 MHz linear array transducer (MyLab 30 VET Gold, ESAOTE, Genoa, Italy) confirmed the absence of reproductive tract disorders. For the experiment, 49 heifers were selected. The estrus was synchronized using the standard procedure of two 5 mg i.m. injections of PGF2α analogue (dinoprost, Dinolytic; Zoetis, Ottignies-Louvain la Neuve, Belgium) with an interval of 11–14 days, as recommended by the vendor. The animals were observed three times a day for signs of estrus activity. Standing heat occurred approximately 72 h after the second dose of the PGF2α analogue. The onset of *estrus* was considered as day 0 of the estrous cycle. To confirm phases of the estrous cycle, the plasma P_4_ concentration was measured. Blood samples were taken from the jugular vein just before slaughtering, i.e., on days 0, 2, 5, 8, 12, 15, 17, 19 and 21 of the estrous cycle. All blood samples were collected into 10 mL ethylenediaminetetraacetic acid heparinized vacutainers (Becton Dickinson Vacutainer Systems, Plymouth, UK). Samples were held in ice until centrifuged at 1500 g at 4 °C for 15 min. Next, plasma was extracted and stored in sterile 7 mL vials at −20 °C until assay using a radioimmunoassay (RIA) [26]. The concentration of P_4_ (ng/mL) in the collected samples during the selected days of the estrous cycle was as follows: day 0—0.38 ± 0.09 (mean ± SEM); day 2—0.069 ± 0.15; day 5—3.77 ± 0.19; day 8—5.96 ± 0.50; day 12—6.94 ± 0.8; day 15—5.70 ± 0.58; day 17—3.08 ± 0.88; day 19—1.76 ± 0.6; day 21—0.69 ± 0.18, as previously described [38]. The estrous cycle phase was additionally confirmed post-mortem through macroscopic observation of the ovary and uterine features according to a previous report [39].

Corpora lutea for experiments were obtained from ovaries and separated from surrounding tissues. For Experiments 1 and 2, CLs were collected on days 2–3 (early luteal phase I, *n* = 8), 5–6 (early luteal phase II, *n* = 8), 8–12 (mid-luteal phase, *n* = 8), 15–17 (late-luteal phase, *n* = 8) and 19–21 (CL regression phase, *n* = 8) of the estrous cycle. Each CL was divided into three parts. For in vitro study (Experiment 3), CLs were obtained on days 15–17 (late-luteal phase, *n* = 9), knowing that luteolysis in cows occurs between days 15 and 17 of the estrous cycle [16]. The animals were culled for economic reasons and as part of herd replacement. All procedures were approved by The Local Animal Care and Use Committee, Olsztyn, Poland (agreement no. 83/2012/N).

### 2.2. Experimental Procedures

#### 2.2.1. Experiment 1: Immunolocalization of PPARα, PPARδ and PPARγ in the Bovine Corpus Luteum

The CL (*n* = 40) samples were fixed for 24 h in 4% paraformaldehyde (PFA) in 0.1 M phosphate-buffered saline (PBS; pH 7.4). Next, they were washed with PBS, dehydrated in a graded ethanol series and embedded in paraffin. Tissue samples were cut at 4 µm thickness with a rotary microtome (HistoCore AVTO-CVT-U 2040, Leica, Germany) and mounted on SuperFrost Plus microscope slides (Menzel-Glaser, Braunschweig, Germany).

#### 2.2.2. Experiment 2: mRNA Expression and Tissue Concentration of PPARα, PPARδ and PPARγ in the Bovine Corpus Luteum throughout the Estrous Cycle

The CL (*n* = 40) samples were transferred into cryo-tubes, frozen rapidly in liquid nitrogen and stored at −80 °C until further processing. The mRNA expression of *PPARA*, *PPARD* and *PPARG* in the bovine CL was measured through quantitative PCR (qPCR). The concentration of PPARα, PPARδ and PPARγ in the CL tissue homogenates was measured using ELISA.

#### 2.2.3. Experiment 3: The Involvement of PPARα, PPARδ and PPARγ in PGF_2α_-Induced Processes Related to Luteolysis in the Bovine Corpus Luteum—In Vitro Study

##### Experiment 3.1: The Effect of PGF_2α_ on PPARα, PPARδ and PPARγ mRNA Expression in the Bovine Corpus Luteum

For in vitro tissue culture, bovine CLs were collected on days 15–17 of the estrous cycle (*n* = 9), knowing that luteolysis in cows occurs between days 15 and 17 of the estrous cycle [16]. Corpora lutea were obtained within 5–10 min after slaughter and transported on ice within 30–40 min to the laboratory. The CL explants (30 mg) were placed in culture vials containing 2 mL of Dulbecco’s Modified Eagle’s Medium (DMEM; PANBiotech GmbH, Aidenbach, Germany, P04-05551) supplemented with 0.1% (*w*/*v*) bovine serum albumin (BSA; Sigma-Aldrich, Saint Louis, MO, USA, A2058) and antibiotics (Penicillin-Streptomycin; penicillin 10,000 units with streptomycin 10 mg/mL, Sigma-Aldrich, Saint Louis, MO, USA, P4333), and preincubated in vitro in a humidified atmosphere of air with 5% CO_2_ at 37.5 °C for 2 h. Next, explants were cultured for 24 h with PGF_2α_ (10^−6^ M; Sigma-Aldrich, Saint Louis, MO, USA, P5069) without medium exchange. Experimental groups were marked as follows: C—control group (untreated CL explants); P—CL explants stimulated with PGF_2α_. The concentration of PGF_2α_ and the duration of tissue stimulation were selected based on a preliminary study (data not shown) and previous reports [28,40]. Tissue explants were frozen at −80 °C until the determination of the mRNA expression of *PPARA*, *PPARD* and *PPARG* using qPCR.

##### Experiment 3.2: The Effect of PGF_2α_ on PPAR-Mediated P_4_ Release and mRNA Expression of Steroidogenic Enzymes and Those Responsible for AA Metabolism, and Selected Factors Mediating Luteolysis in the Bovine Corpus Luteum

**Scheme 1:**
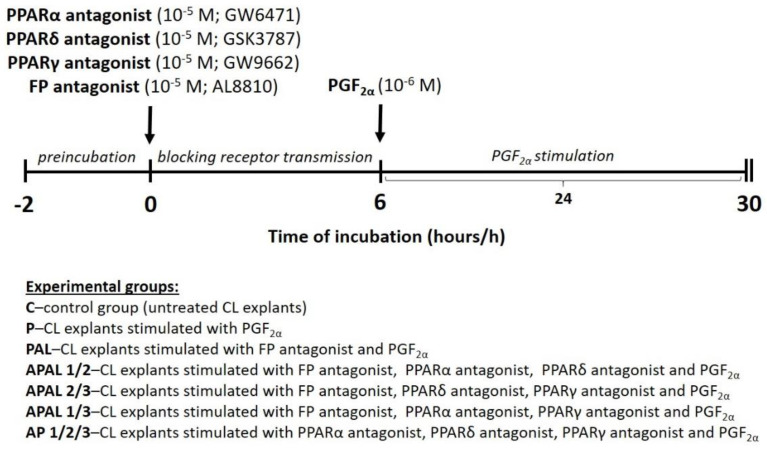
Scheme 1 of the estrous cycle (*n* = 9). The CL explants were preincubated in vitro for 2 h. Next, explants were cultured in the presence of PPAR antagonists: PPARα antagonist (10^−5^ M, GW6471; Sigma-Aldrich, Saint Louis, MO, USA, G5045), PPARδ antagonist (10^−5^ M, GSK3787; Sigma-Aldrich, Saint Louis, MO, USA, G7423) and PPARγ antagonist (10^−5^ M, GW9662; Cayman Chemical, Ann Arbor, MI, USA, 70785), in combination with or without PGF_2α_ receptor (FP) antagonist (10^−5^ M, AL8810; Sigma-Aldrich, Saint Louis, MO, USA, A3846) to block receptor action for 6 h, and then CL explants were stimulated with PGF_2α_ (10^−6^ M; Sigma-Aldrich, Saint Louis, MO, USA, P5069) for a further 24 h without medium exchange. Experimental groups were marked as follows: **C**—control group (untreated CL explants); **P**—CL explants stimulated with PGF_2α_; **PAL**—CL explants stimulated with FP antagonist and PGF_2α_; **APAL 1/2**—CL explants stimulated with FP antagonist, PPARα antagonist, PPARδ antagonist and PGF_2α_; **APAL 2/3**—CL explants stimulated with FP antagonist, PPARδ antagonist, PPARγ antagonist and PGF_2α_; **APAL 1/3**—CL explants stimulated with FP antagonist, PPARα antagonist, PPARγ antagonist and PGF_2α_; and **AP 1/2/3**—CL explants stimulated with PPARα antagonist, PPARδ antagonist, PPARγ antagonist and PGF_2α_. After incubation, the culture medium was frozen at −20 °C until further determination using RIA. Tissue explants were frozen at −80 °C until further analysis using qPCR.

For in vitro tissue culture, bovine CLs were collected on days 15–17 of the estrous cycle (*n* = 9). Corpora lutea were obtained within 5–10 min after slaughter and transported on ice within 30–40 min to the laboratory. The CL explants (30 mg) were placed in culture vials containing 2 mL of Dulbecco’s Modified Eagle’s Medium (DMEM; PANBiotech GmbH, Aidenbach, Germany, P04-05551) supplemented with 0.1% (*w*/*v*) bovine serum albumin (BSA; Sigma-Aldrich, Saint Louis, MO, USA, A2058) and antibiotics (Penicillin-Streptomycin; penicillin 10,000 units with streptomycin 10 mg/mL, Sigma-Aldrich, Saint Louis, MO, USA, P4333), and preincubated in vitro in a humidified atmosphere of air with 5% CO_2_ at 37.5 °C for 2 h. Next, explants were cultured according to the adopted scheme (Scheme 1) in the presence of PPAR antagonists: PPARα antagonist (10^−5^ M, GW6471; Sigma-Aldrich, Saint Louis, MO, USA, G5045), PPARδ antagonist (10^−5^ M, GSK3787; Sigma-Aldrich, Saint Louis, MO, USA, G7423) and PPARγ antagonist (10^−5^ M, GW9662; Cayman Chemical, Ann Arbor, MI, USA, 70785), in combination with or without PGF_2α_ receptor (FP) antagonist (10^−5^ M, AL8810; Sigma-Aldrich, Saint Louis, MO, USA, A3846) to block receptor action for 6 h, and then CL explants were stimulated with PGF_2α_ (10^−6^ M; Sigma-Aldrich, Saint Louis, MO, USA, P5069) for a further 24 h without medium exchange. Experimental groups were marked as follows: C—control group (untreated CL explants); P—CL explants stimulated with PGF_2α_; PAL—CL explants stimulated with FP antagonist and PGF_2α_; APAL 1/2—CL explants stimulated with FP antagonist, PPARα antagonist, PPARδ antagonist and PGF_2α_; APAL 2/3—CL explants stimulated with FP antagonist, PPARδ antagonist, PPARγ antagonist and PGF_2α_; APAL 1/3—CL explants stimulated with FP antagonist, PPARα antagonist, PPARγ antagonist and PGF_2α_; and AP 1/2/3—CL explants stimulated with PPARα antagonist, PPARδ antagonist, PPARγ antagonist and PGF_2α_.

The concentrations of factors and the duration of tissue stimulation were selected based on a preliminary study (data not shown) and previous reports [28,40,41]. After incubation, the culture medium was transferred to tubes containing 5% EDTA and 1% acetylsalicylic acid solution (pH 7.4). It was frozen at −20 °C until the determination of P_4_ by RIA. Tissue explants were frozen at −80 °C until the determination of mRNA expression of (1) steroidogenic enzymes: *StAR*, *P450scc* and *HSD3B1*; (2) enzymes responsible for AA metabolism: *PTGS2* and *PTGFS*; (3) selected factors mediating luteolysis: *TNFα*, *TNFRSF1A*, *TNFRSF1B* and *iNOS* using qPCR.

### 2.3. Total RNA Isolation and cDNA Synthesis

Total RNA was extracted from CL tissue (30 mg) using TRI-Reagent (Sigma-Aldrich, Saint Louis, MO, USA, T9424), according to the manufacturer’s instructions. The RNA content and purity were assessed using a NanoDrop 1000 spectrophotometer (Thermo Fisher Scientific, Wilmington, DE, USA, ND-1000). The absorbance ratio of 260/280 was approx. 2.0, and the absorbance ratio of 260/230 ranged between 1.8 and 2.2. To remove genomic DNA contamination, RNA samples were treated with DNase I, Amplification Grade (Sigma-Aldrich, Saint Louis, MO, USA, AMPD1-KT). One microgram (µg) of total RNA was reverse transcribed to cDNA using the High-Capacity cDNA Reverse Transcription Kit for RT-PCR (Applied Biosystems, Foster City, CA, USA, 4368814) containing MultiScribe^TM^ Reverse Transcriptase with random primers, dNTP mixture, MgCl_2_, RNase Inhibitor and nuclease-free H_2_O, according to the manufacturer’s instructions. The reverse transcription conditions were as follows: 25 °C for 10 min, 37 °C for 120 min, 85 °C for 5 min and 4 °C for 1 h. The obtained cDNA was stored at −20 °C until qPCR quantification.

### 2.4. qPCR Quantification

The qPCR experiments were performed according to the MIQE guidelines [42], as previously described [38]. The ABI 7900 HT sequence detection system (Applied Biosystems, Foster City, CA, USA) was used with the SensiFAST SYBR Hi-ROX Kit (Bioline Reagents, London, UK, BIO-92002). The total volume of the reaction was 10 µL and contained 5 µL of SensiFAST SYBR Hi-ROX Master Mix, 1 µL each of forward and reverse primers (0.5 µM) and 3 µL of reverse-transcribed cDNA (10 ng). The primer sequences for determining the mRNA expression of reference and target genes were chosen based on scientific reports, i.e., reference genes: *glyceraldehyde-3-phosphate dehydrogenase* (*GAPDH*) [38,43], *beta-actin* (*ACTB*) [38,43] and *18S ribosomal RNA* (*RN18S1*) [38,43], and target genes: *PPARα* (*PPARA*) [38,43], *PPARδ* (*PPARD*) [38,43], *PPARγ* (*PPARG*) [38,43], *steroidogenic acute regulatory protein* (*StAR*) [20], *cytochrome P450 family 11 subfamily A member 1* (*P450scc*) [20], *hydroxy-delta-5-steroid dehydrogenase, 3 β- and steroid delta-isomerase 1* (*HSD3B1*) [20], *prostaglandin-endoperoxide synthase 2* (*PTGS2*) [44], *prostaglandin F_2α_ synthase* (*PTGFS*) [44], *tumor necrosis factor α* (*TNFα*) [19], *tumor necrosis factor receptor superfamily member 1A* (*TNFRSF1A*) [19], *tumor necrosis factor receptor superfamily member 1B* (*TNFRSF1B*) [19] and *inducible nitric oxide synthase* (*iNOS*) [45]. All primers were synthesized by Sigma-Aldrich (Custom DNA Oligos, Sigma-Aldrich, Saint Louis, MO, USA). The primer sequences, GenBank accession numbers and the size of the products are presented in Table 1.

The samples were run in duplicate. qPCR was carried out as follows: initial enzyme activation step (95 °C for 2 min), followed by 40 cycles of denaturation (95 °C for 5 s) and annealing (60 °C for 20 s). To ensure single product amplification, melting curves were obtained after each PCR reaction by gradually increasing the temperature from 50–95 °C. To confirm that products were free from primer-dimers and genomic DNA contamination, respectively, control reactions lacking a template or primers were performed. The stability of the reference genes was determined in the NormFinder program [46]. The RT-qPCR results were calculated using the ΔΔC_t_ method described by Livak and Schmittgen [47]. The gene expression data in our study are expressed relative to the best combination of two reference genes, as a ratio of target genes to the *GAPDH/RN18S1*, and are presented as arbitrary units.

### 2.5. Immunohistochemistry

Immunostaining was carried out according to a published protocol [38]. The sections were deparaffinized and rehydrated. To block endogenous peroxidase activity, they were treated for 20 min with 0.3% hydrogen peroxide in methanol. Then, the slides were washed in 0.1 M PBS. Depending on the host of the used primary antibodies, sections were blocked with 10% normal goat serum (Sigma-Aldrich, Madison, WI, USA, G9023) or 5% BSA (Sigma-Aldrich, Saint Louis, MO, USA, A2058) for 60 min at RT (approx. 23 °C, RT) to block nonspecific sites, and then incubated overnight at RT with primary antibodies, including a 1:50 dilution of anti-PPARα (polyclonal antibody; host—rabbit; reactivity—bovine; Cayman Chemical, Ann Arbor, MI, USA, 101710), a 1:50 dilution of anti-PPARδ (polyclonal antibody; host—goat; reactivity—bovine; Abcam, Cambridge, UK, ab21209) and a 1:50 dilution of anti-PPARγ (polyclonal antibody; host—rabbit; reactivity—bovine; Cayman Chemical, Ann Arbor, MI, USA, 101700). After washing in PBS, sections were incubated for 60 min at RT with a 1:500 dilution of secondary biotinylated anti-rabbit (Abcam, Cambridge, UK, PK-6101) or anti-goat (Abcam, Cambridge, UK, PK-6105) antibodies (Vectastain ABC Kit; Vector Laboratories, Burlingame, CA, USA, BA 9200). Slides were washed, incubated for 45 min with ABC reagent in PBS and washed again. The proteins were visualized by incubating the sections for 2 to 3 min in 0.3 mg/mL 3,3′-diaminobenzidine tetrahydrochloride (Sigma-Aldrich, Saint Louis, MO, USA, D5637) in 0.01% hydrogen peroxide in Tris-buffered saline (pH 7.2). Hematoxylin counterstaining was used to visualize cell nuclei, and to obtain contrast. Next, sections were dehydrated and cover-slipped with DPX mounting medium (PanReac, Barcelona, Spain, 255254). Negative controls were obtained by replacing the primary antibody with PBS. Positive IHC staining was assessed as a characteristic brown staining. Observations were made and photographs were taken using a light microscope (Nikon FXA, Tokyo, Japan).

### 2.6. PPARα, PPARβ/δ and PPARγ Determinations

Measurements of PPAR concentration in the bovine CL tissue homogenates (100 mg) were performed using commercially available ELISA kits, according to the manufacturer’s instructions. Initially, CL tissue was rinsed with 1X PBS to remove excess blood, homogenized in 20 mL of 1X PBS and stored overnight at ≤−20 °C. Then, two freeze–thaw cycles were performed to break the cell membranes, and homogenates were centrifuged for 5 min at 5000× *g*. Next, the supernatant was removed and assayed immediately.

The determination of PPARα tissue concentration was performed using a Bovine Peroxisome proliferator-activated receptor α ELISA Kit (MyBioSource, San Diego, CA, USA, MBS748844). The standard curve ranged from 50 pg/mL to 1000 pg/mL. The intra- and inter-assay CV values averaged <8% and <10%, respectively. To evaluate the PPARδ tissue concentration Bovine Peroxisome proliferator-activated receptor δ ELISA Kit (MyBioSource, San Diego, CA, USA, MBS9924325) was used. The standard curve ranged from 78 pg/mL to 5000 pg/mL. The intra- and inter-assay CV values averaged <8% and <10%, respectively. The determination of the PPARγ tissue concentration was performed using Bovine peroxisome proliferator-activated receptor γ ELISA Kit (Wuhan EIAab Science Co., Wuhan, China, E0886b). The standard curve ranged from 0.78 ng/mL to 50 ng/mL. The intra- and inter-assay CV values averaged <10% and <12%, respectively.

### 2.7. Progesterone Determination

Measurements of P_4_ were performed in blood plasma and medium by direct radioimmunoassay (RIA; DIASource ImmunoAssays S.A., Nivelles, Belgium, KIP1458). The standard curve ranged from 0.12–36 ng/mL. The effective dose for 50% inhibition (ED 50) of the assay was 0.05 ng/mL. The intra- and inter-assay coefficients of variation (CV) were 6.5% and 8.6%, respectively.

### 2.8. Statistical Analysis

For each statistical analysis, a Gaussian distribution was tested using the D’Agostino and Pearson normality test (GraphPad Software version 9; GraphPad, San Diego, CA, USA). The Shapiro–Wilk test was performed to test the normality of the data. In Experiment 2, the mRNA expression profiles of PPARs were presented in arbitrary units as the ratio of expression of the target genes to the mean of the best combination of two reference genes, including *GAPDH* and *RN18S1*, and the PPAR tissue concentration was expressed in pg/g tissue. The data obtained from tissue culture were expressed as a fold change or % of control. In Experiment 2, statistical differences between groups throughout the estrous cycle were determined using the nonparametric one-way ANOVA Kruskal–Wallis followed by Dunn’s multiple comparisons test. In Experiment 3.1, statistical differences between control and PGF_2α_-treated explants were determined using the nonparametric Mann–Whitney U test. In Experiment 3.2, data were analyzed using nonparametric one-way ANOVA Kruskal–Wallis followed by Dunn’s multiple comparisons test. As it would be difficult to indicate in one figure all the correlations found between all experimental groups, only changes between the PGF_2α_-treated group (*p*) compared to the other experimental groups (treated with FP and PPAR antagonists: PAL, APAL 1/2, APAL 2/3, APAL 1/3 and AP 1/2/3) are marked in Figures according to the main objectives of the study. Other correlations (i.e., control group versus other experimental groups) are presented in Supplementary Figures. The data are shown as the mean ± SEM. The results were considered significantly different at *p* < 0.05.

## 3. Results

### 3.1. Immunolocalization of PPARα, PPARδ and PPARγ in the Bovine Corpus Luteum

**Figure 1:**
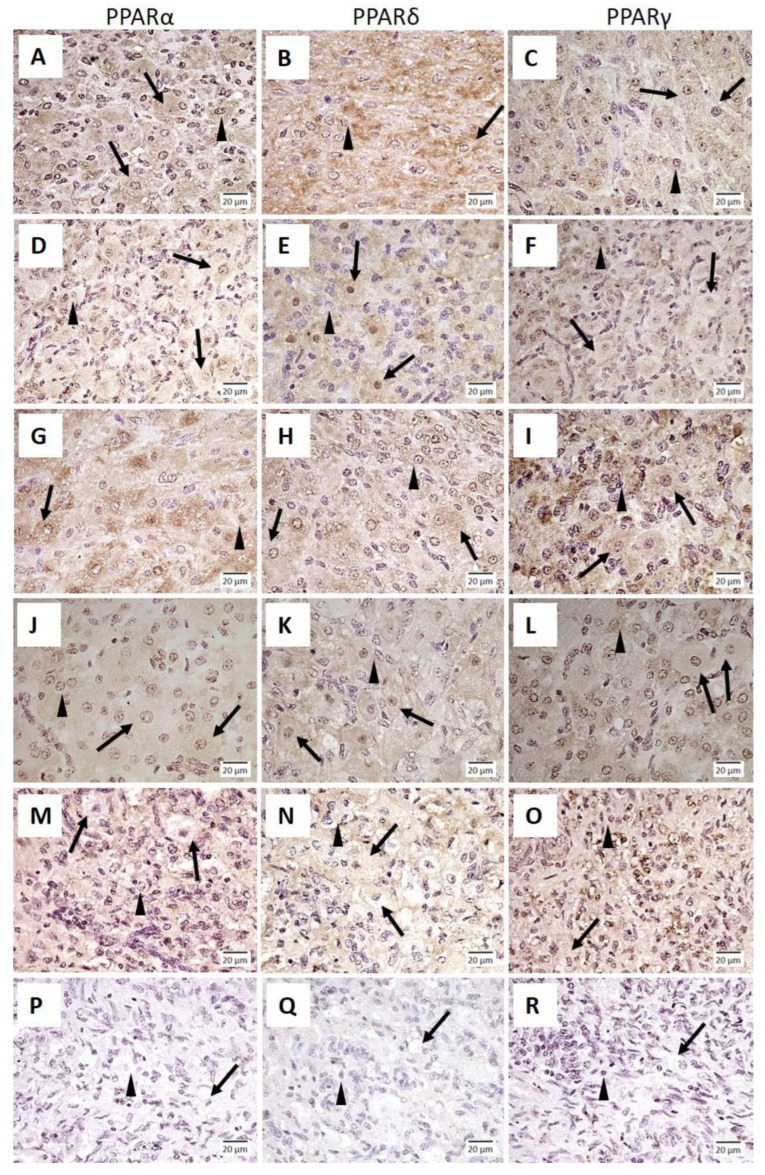
Representative images of immunohistochemical staining of PPARα (first column), PPARδ (second column) and PPARγ (third column) in the bovine corpus luteum (CL) during early luteal I (days 2–3; (**A**–**C**)), early luteal II (days 5–6; (**D**–**F**)), mid-luteal (days 8–12; (**G**–**I**)), late-luteal (days 15–17; (**J**–**L**)) and CL regression (days 19–21; (**M**–**O**)) phases of the estrous cycle. The negative controls (no Ab) are shown in (**P**–**R**) images. Positive immunohistochemical staining was assessed as brown staining. Arrows—large luteal cells of the CL; arrowheads—small luteal cells of the CL. Bar = 20 μm.

Immunohistochemistry revealed the localization of PPARα, PPARδ and PPARγ in the examined bovine CL during the estrous cycle. Each PPAR isoform was detected and localized in the perinuclear cytoplasm and nuclei of luteal cells at early luteal I (days 2–3; Figure 1A–C), early luteal II (days 5–6; Figure 1D–F), mid-luteal (days 8–12; Figure 1G–I) and late-luteal (days 15–17; Figure 1J–L) phases of the estrous cycle. A decreased immunoreactivity of PPARs in the nuclei of luteal cells was observed in the CL regression phase (days 19–21; Figure 1M–O) of the estrous cycle. Figure 1 shows representative pictures of immunohistochemical staining for PPARα, PPARδ and PPARγ in the bovine CL throughout the estrous cycle.

### 3.2. mRNA Expression and Tissue Concentration of PPARα, PPARδ and PPARγ in the Bovine Corpus Luteum throughout the Estrous Cycle

**Figure 2:**
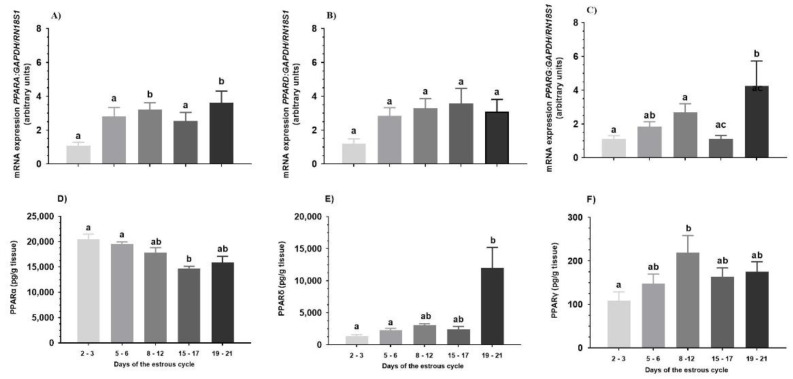
The mRNA expression of *PPARA* (**A**), *PPARD* (**B**) and *PPARG* (**C**), and tissue concentration of PPARα (**D**), PPARδ (**E**) and PPARγ (**F**) in the bovine CL on days 2–3, 5–6, 8–12, 15–17 and 19–21 of the estrous cycle. The mRNA expression profiles are presented in arbitrary units as the ratio of expression of the target genes to the mean of the best combination of two reference genes, namely, glyceraldehyde-3-phosphate dehydrogenase (GAPDH) and ribosomal 18S RNA (RN18S1). The concentration is expressed as pg/g tissue. Presented results are the mean ± SEM from 8 animals. The superscript letters “a, b, c” indicate the statistical differences between groups, as determined by nonparametric one-way ANOVA Kruskal–Wallis followed by Dunn’s multiple comparisons test.

The mRNA expression of PPARA in the bovine CL was upregulated on days 8–12 (*p* < 0.05; Figure 2A) and 19–21 (*p* < 0.05; Figure 2A) compared to days 2–3 of the estrous cycle. There were no significant differences in the mRNA expression of PPARD in the CL throughout the estrous cycle (*p* > 0.05; Figure 2B). The mRNA expression of PPARG in the bovine CL was upregulated on days 19–21 relative to days 2–3 (*p* < 0.05; Figure 2C) and 15–17 (*p* < 0.05; Figure 2C) of the estrous cycle.

The concentration of PPARα in the bovine CL was lower on days 15–17 compared to days 2–3 and 5–6 of the estrous cycle (*p* < 0.05; Figure 2D). Additionally, the concentration of PPARδ in the CL was significantly higher on days 19–21 compared to days 2–3 and 5–6 of the estrous cycle (*p* < 0.0001; Figure 2E). The concentration of PPARγ was higher on days 8–12 compared to days 2–3 (*p* < 0.05; Figure 2F) of the estrous cycle.

### 3.3. The Involvement of PPARα, PPARδ and PPARγ in PGF_2α_-Induced Processes Related to Luteolysis in the Bovine Corpus Luteum—In Vitro Study

#### 3.3.1. The Effect of PGF_2α_ on PPARα, PPARδ and PPARγ mRNA Expression in the Bovine Corpus Luteum

**Figure 3:**
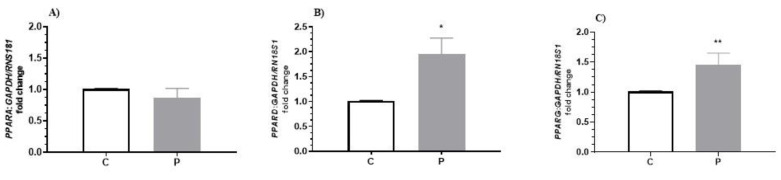
The mRNA expression of *PPARA* (**A**), *PPARD* (**B**) and *PPARG* (**C**) in the bovine CL explants after 24 h of incubation with PGF_2α_ (10^−6^ M) on days 15–17 of the estrous cycle. The mRNA expression profiles are presented as a fold change. Presented results are the mean ± SEM from 9 animals. The asterisks indicate statistical differences (* *p* < 0.05, ** *p* < 0.01) between control (**C**) and PGF_2α_-treated (**P**) explants, as determined by the nonparametric Mann–Whitney U test.

In bovine PGF_2α_-treated CL explants (P), the mRNA expression of *PPARD* (*p* < 0.05; Figure 3B) and *PPARG* (*p* < 0.01; Figure 3C) was upregulated compared to the corresponding control (C; untreated CL explants). There were no significant differences in *PPARA* mRNA expression in the CL explants after 24 h of PGF_2α_ treatment relative to the control explants (*p* > 0.05; Figure 3A).

#### 3.3.2. The Effect of PGF_2α_ on PPAR-Mediated P_4_ Release and mRNA Expression of Steroidogenic Enzymes and Those Responsible for AA Metabolism, and Selected Factors Mediating Luteolysis in the Bovine Corpus Luteum

**Figure 4:**
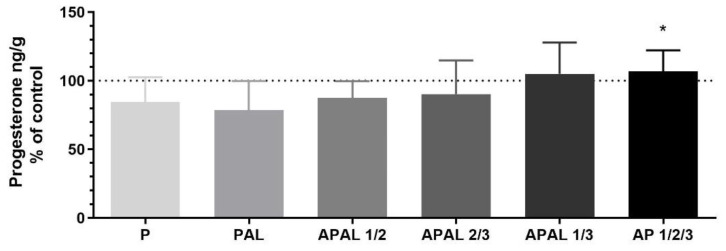
The effect of inhibition of PPARα, PPARδ, PPARγ and PGF_2α_ receptor (FP) in the bovine PGF_2α_-treated CL explants on progesterone (P_4_) secretion on days 15–17 of the estrous cycle. The results are presented as a % of the control group (untreated CL explants, dotted line). Presented results are the mean ± SEM from 9 animals. The asterisks indicate statistical differences between the PGF_2__α_-only treatment (P) group versus the other experimental groups (* *p* < 0.05), as determined by nonparametric one-way ANOVA Kruskal–Wallis followed by Dunn’s multiple comparisons test. The groups are marked as follows: **P**—CL explants stimulated with PGF_2α_ (10^−6^ M); **PAL**—CL explants stimulated with FP antagonist (10^−5^ M) and PGF_2α_ (10^−6^ M); **APAL 1/2**—CL explants stimulated with FP antagonist (10^−5^ M), PPARα antagonist (10^−5^ M), PPARδ antagonist (10^−5^ M) and PGF_2α_ (10^−6^ M); **APAL 2/3**—CL explants stimulated with FP antagonist (10^−5^ M), PPARδ antagonist (10^−5^ M), PPARγ antagonist (10^−5^ M) and PGF_2α_ (10^−6^ M); **APAL 1/3**—CL explants stimulated with FP antagonist (10^−5^ M), PPARα antagonist (10^−5^ M), PPARγ antagonist (10^−5^ M) and PGF_2α_ (10^−6^ M); **AP 1/2/3**—CL explants stimulated with PPARα antagonist (10^−5^ M), PPARδ antagonist (10^−5^ M), PPARγ antagonist (10^−5^ M) and PGF_2α_ (10^−6^ M).

The concentration of P_4_ in the culture medium after 24 h stimulation with PGF_2α_ decreased in the P (*p* < 0.05), PAL (*p* < 0.05) and APAL 1/2 (*p* < 0.05) groups compared to the control group (C; untreated CL explants; data not shown; see Supplementary Figure S1). Although there were no significant differences between the PGF_2α_-treated group and the PAL, APAL 1/2, APAL 2/3 and APAL 1/3 groups (*p* > 0.05; P group versus PAL, APAL 1/2, APAL 2/3 and APAL 1/3; Figure 4), pre-treatment with the PPARα, PPARδ and PPARγ antagonist (AP 1/2/3) groups reversed the PGF_2α_ inhibitory effect of P_4_ secretion (*p* < 0.05; P group versus AP 1/2/3; Figure 4).

**Figure 5:**
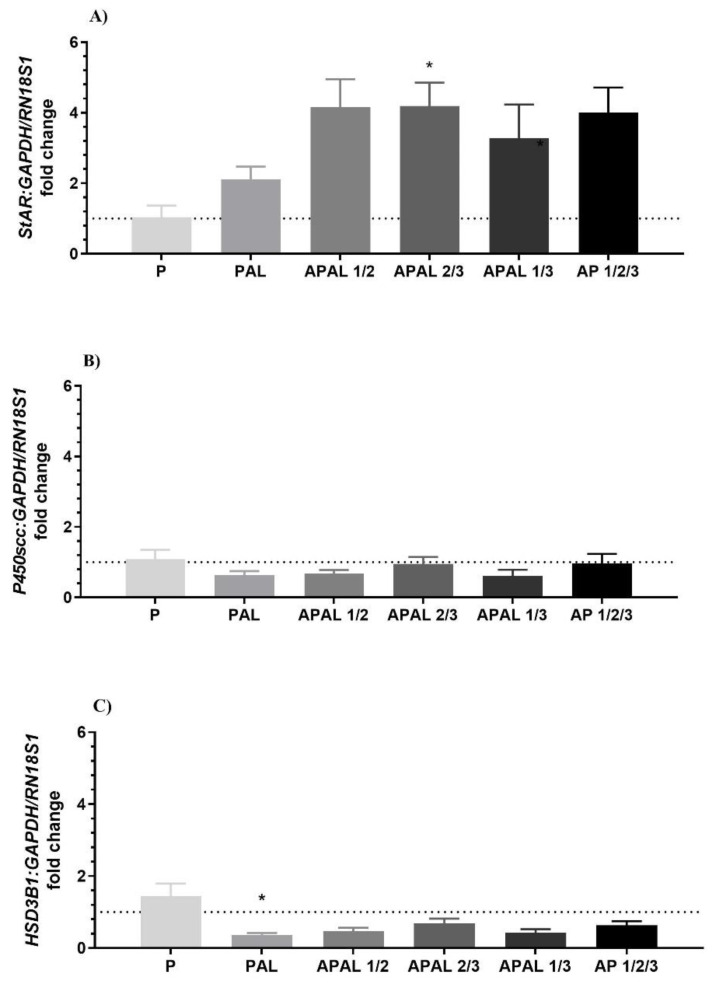
The effect of inhibition of PPARα, PPARδ, PPARγ and PGF_2α_ receptor (FP) in the bovine PGF_2α_-treated CL explants on mRNA expression of *StAR* (**A**), *P450scc* (**B**), and *HSD3B1* (**C**) on days 15–17 of the estrous cycle. The results are presented as a fold change in the control group (untreated CL explants, dotted line). Presented results are the mean ±SEM from 9 animals. The asterisks indicate statistical differences between the PGF_2α_-only treatment (P) group versus other experimental groups (* *p* < 0.05), as determined by nonparametric one-way ANOVA Kruskal–Wallis followed by Dunn’s multiple comparisons test. The groups are marked as follows: **P**—CL explants stimulated with PGF_2α_ (10^−6^ M); **PAL**—CL explants stimulated with FP antagonist (10^−5^ M) and PGF_2α_ (10^−6^ M); **APAL 1/2**—CL explants stimulated with FP antagonist (10^−5^ M), PPARα antagonist (10^−5^ M), PPARδ antagonist (10^−5^ M) and PGF_2α_ (10^−6^ M); **APAL 2/3**—CL explants stimulated with FP antagonist (10^−5^ M), PPARδ antagonist (10^−5^ M), PPARγ antagonist (10^−5^ M) and PGF_2α_ (10^−6^ M); **APAL 1/3**—CL explants stimulated with FP antagonist (10^−5^ M), PPARα antagonist (10^−5^ M), PPARγ antagonist (10^−5^ M) and PGF_2α_ (10^−6^ M); **AP 1/2/3**—CL explants stimulated with PPARα antagonist (10^−5^ M), PPARδ antagonist (10^−5^ M), PPARγ antagonist (10^−5^ M) and PGF_2α_ (10^−6^ M).

In addition, the mRNA expression of *StAR* in the bovine CL explants after 24 h PGF_2α_ stimulation increased in APAL 1/2 (*p* < 0.05), APAL 2/3 (*p* < 0.05) and AP 1/2/3 (*p* < 0.05) groups relative to the control explants (data not shown; see Supplementary Figure S2A). Moreover, the differences in the mRNA expression of *StAR* were observed in the APAL 2/3 group in comparison with the PGF_2α_-treated group (*p* < 0.05; P group versus APAL 2/3) (Figure 5A).

There were no significant differences in the *P450scc* mRNA expression in the bovine CL explants after 24 h of PGF_2α_ stimulation in all experimental groups relative to the control group (*p* > 0.05; data not shown; see Supplementary Figure S2B). Additionally, there were no significant differences in the *P450scc* mRNA expression after 24 h of PGF_2α_ stimulation among all experimental groups (*p* > 0.05; Figure 5B).

On the other hand, the mRNA expression of *HSD3B1* was downregulated in the PAL (*p* < 0.01), APAL 1/2 (*p* < 0.05) and APAL 1/3 (*p* < 0.05) groups compared to the control group (data not shown; see Supplementary Figure S2C). Moreover, significant differences in *HSD3B1* were noted in the PAL group compared to the PGF_2α_-treated group (*p* < 0.05; P group versus PAL; Figure 5C).

**Figure 6:**
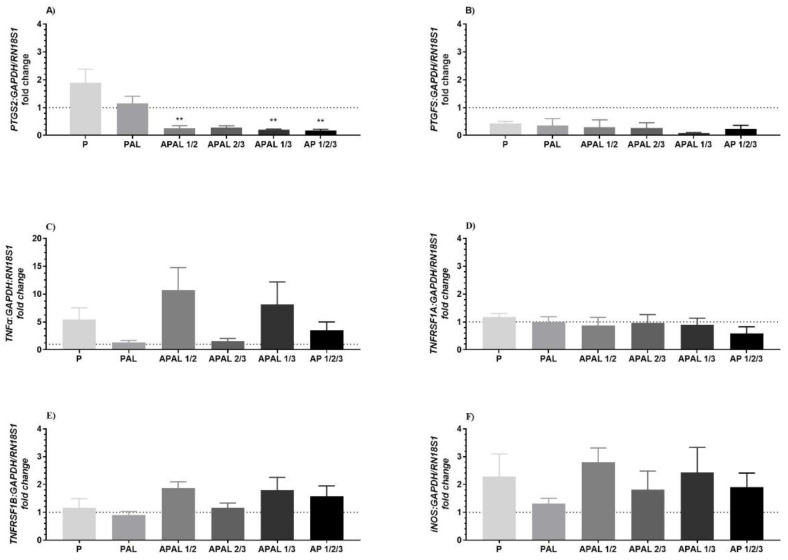
The effect of inhibition of PPARα, PPARδ, PPARγ and PGF_2α_ receptor (FP) in the bovine PGF_2α_-treated CL explants on mRNA expression of *PTGS2* (**A**), *PGFS* (**B**), *TNFα* (**C**), *TNFRSF1A* (**D**), *TNFRSF1B* (**E**) and *iNOS* (**F**) on days 15–17 of the estrous cycle. The results are presented as a fold change in the control group (untreated CL explants, dotted line). Presented results are the mean ± SEM from 9 animals. The asterisks indicate statistical differences between the PGF_2α_-only treatment (P) group versus the other experimental groups (** *p* < 0.01) as determined by nonparametric one-way ANOVA Kruskal–Wallis followed by Dunn’s multiple comparisons test. The groups are marked as follows: **P**—CL explants stimulated with PGF_2α_ (10^−6^ M); **PAL**—CL explants stimulated with FP antagonist (10^−5^ M) and PGF_2α_ (10^−6^ M); **APAL 1/2**—CL explants stimulated with FP antagonist (10^−5^ M), PPARα antagonist (10^−5^ M), PPARδ antagonist (10^−5^ M) and PGF_2α_ (10^−6^ M); **APAL 2/3**—CL explants stimulated with FP antagonist (10^−5^ M), PPARδ antagonist (10^−5^ M), PPARγ antagonist (10^−5^ M) and PGF_2α_ (10^−6^ M); **APAL 1/3**—CL explants stimulated with FP antagonist (10^−5^ M), PPARα antagonist (10^−5^ M), PPARγ antagonist (10^−5^ M) and PGF_2α_ (10^−6^ M); **AP 1/2/3**—CL explants stimulated with PPARα antagonist (10^−5^ M), PPARδ antagonist (10^−5^ M), PPARγ antagonist (10^−5^ M) and PGF_2α_ (10^−6^ M).

The mRNA expression of *PTGS2* in the bovine CL explants after 24 h PGF_2α_ stimulation was upregulated in the P (*p* < 0.05) group compared to the control group (Figure 6A), while it was downregulated in the APAL 1/2 (*p* < 0.05), APAL 2/3 (*p* < 0.05), APAL 1/3 (*p* < 0.05) and AP 1/2/3 (*p* < 0.01) groups relative to the control group (data not shown; see Supplementary Figure S3A). Additionally, in the APAL 1/2, APAL 1/3 and AP 1/2/3 groups, the PGF_2α_ stimulatory effect on *PTGS2* mRNA expression was reversed (*p* > 0.05; P group versus APAL 1/2, APAL 1/3 and AP 1/2/3; Figure 6A).

The mRNA expression of *PTGFS* in the bovine CL explants was downregulated in the APAL 1/3 (*p* < 0.001) and AP 1/2/3 (*p* < 0.01) groups compared to the corresponding control group (data not shown; see Supplementary Figure S3B). Additionally, there were no differences among the PGF_2α_-treated group and the PAL, APAL 1/2, APAL 2/3, APAL 1/3 and AP 1/2/3 groups (*p* > 0.05; P group versus PAL, APAL 1/2, APAL 2/3, APAL 1/3 and AP 1/2/3 groups; Figure 6B).

The mRNA expression of *TNFα* in the bovine CL explants after 24 h of PGF_2α_ stimulation was upregulated in the P (*p* < 0.05) and APAL 1/2 (*p* < 0.01) groups compared to the corresponding control group (data not shown; see Supplementary Figure S3C). However, there were no differences among the PGF_2α_-treated group and the PAL, APAL 1/2, APAL 2/3, APAL 1/3 and AP 1/2/3 groups (*p* > 0.05; P group versus PAL, APAL 1/2, APAL 2/3, APAL 1/3 and AP 1/2/3 groups; Figure 6C).

Additionally, *TNFRSF1A* mRNA expression increased in the P group compared to the corresponding control group (*p* < 0.05; data not shown; see Supplementary Figure S3D). There were no differences between the PGF_2α_-treated group and the PAL, APAL 1/2, APAL 2/3, APAL 1/3 and AP 1/2/3 groups (*p* > 0.05; P group versus PAL, APAL 1/2, APAL 2/3, APAL 1/3 and AP 1/2/3 groups; Figure 6D).

*TNFRSF1B* mRNA expression in the bovine CL explants after 24 h PGF_2α_ stimulation was upregulated in the APAL 1/2 group relative to the control group (*p* < 0.05; data not shown; see Supplementary Figure S3E). However, there were no differences among the PGF_2α_-treated group and the PAL, APAL 1/2, APAL 2/3, APAL 1/3 and AP 1/2/3 groups (*p* > 0.05; P group versus PAL, APAL 1/2, APAL 2/3, APAL 1/3 and AP 1/2/3 groups; Figure 6E).

The mRNA expression of *iNOS* in the CL explants increased in the P (*p* < 0.05) and APAL 1/2 (*p* < 0.05) groups relative to the control explants (data not shown; see Supplementary Figure S3F). Moreover, there were no differences among the PGF_2α_-treated group and the PAL, APAL 1/2, APAL 2/3, APAL 1/3 and AP 1/2/3 groups (*p* > 0.05; P group versus PAL, APAL 1/2, APAL 2/3, APAL 1/3 and AP 1/2/3 groups; Figure 6F).

## 4. Discussion

To the best of our knowledge, the present study is the first-ever report that demonstrates differences in PPARα, PPARδ and PPARγ immunodetection and immunolocalization as well as the mRNA expression and tissue concentration in the bovine CL at different luteal stages. Moreover, it shows changes in the expression of PPAR isoforms under the influence of PGF_2α_ and their involvement in PGF_2α_-induced processes related to CL regression.

Our immunohistochemical findings demonstrated the presence of each PPAR isoform in the cytoplasm and nuclei of luteal cells in all investigated phases of the estrous cycle. It is worth noting that in the CL regression phase, the majority of nuclei were immunonegative, which is in accordance with the results obtained in the late CL of rabbits, where PPARγ immunoreactivity in the nuclei of luteal cells was also decreased [48]. The obtained results suggest the participation of all PPAR isoforms in the regulation of the CL lifespan throughout the estrous cycle.

The mRNA and protein expression of PPARα, PPARδ and PPARγ differed depending on the luteal stages. The tissue concentration of PPARα was decreased on days 15–17 compared to days 2–3 of the estrous cycle, which could suggest its potential role in the formation and maintenance of the bovine CL in the early luteal phases of the estrous cycle. In turn, we observed that the PPARδ tissue concentration in the bovine CL was higher on days 19–21 compared to days 2–3 of the estrous cycle, which indicates a possible involvement of this isoform in the processes related to luteolysis and the CL regression. Furthermore, the PPARγ tissue concentration in the bovine CL was higher on days 8–12 compared to days 2–3 of the estrous cycle, which is in line with the study of Lőhrke et al. [36], in which the expression of PPARγ in the bovine luteal cells was detected on day 12 of the estrous cycle. Additionally, the mRNA expression of PPARγ increased in the bovine CL on days 19–21 relative to days 2–3 and 15–17 of the estrous cycle. Therefore, these findings suggest that PPARγ could play a role in both the maintenance and regression of bovine CL. However, further investigation is warranted to study these hypotheses, especially the role of PPAR isoforms in the development and maintenance of bovine CL.

It should be noted here that for PPAR isoforms, it appears that there is a different trend of expression between mRNA and protein data throughout the estrous cycle. In our previous studies [14,38,43], we also observed some discrepancies. This should be explained by the fact that transcription and translation are far from having a linear and simple relationship. According to de Sousa Abreu et al. [49] and Vogel and Marcotte [50], the genome-wide correlation between expression levels of mRNA and protein is notoriously poor, hovering around 40% explanatory power across many studies. This discrepancy is typically attributed to other levels of regulation between transcript and protein products [51]. Different events may uncouple transcription and translation. According to Maier et al. [51], this can arise from the NA secondary structure, regulatory protein, regulatory sRNAs, ribosomal density, ribosome occupancy, etc.

The regulatory events occurring between the stage of the estrous cycle and luteolytic PGF_2α_ acting as a PPAR ligand are poorly understood in cows. It is well known that uterine and ovarian PGs are important factors for regulating reproductive processes during luteolysis in cattle [15,16,17,18,19,20,21,52]. The luteolytic action of PGF_2α_ is mediated by its specific plasma membrane receptor (FP) [53]. Prostaglandin F_2α_ is also an endogenous factor that has been shown to activate PPAR [12]. Additionally, it has been suggested that PPARγ may directly affect the expression of PTGS2, which is a rate-limiting enzyme responsible for PGF_2α_ synthesis [6]. In fact, there is a cyclical relationship between the presence of PGs, activation and/or inhibition of PPAR and feedback to PTGS2 [8]. The data obtained in the present study have shown that PGF_2α_ upregulated the mRNA expression of PPARδ and PPARγ in the bovine CL explants on days 15–17 of the estrous cycle. The results regarding PPARγ are consistent with our previous report [14], in which we observed an increase in PPARγ mRNA expression in bovine endometrial stromal cells under the influence of PGF_2α_ on days 8–12 of the estrous cycle. However, there was no difference in the mRNA expression of PPARδ observed, which differs from the results presented in the CL, where we noted the upregulation of PPARδ mRNA expression. These slight differences may be due to the different luteal phases of the estrous cycle selected for the in vitro experiments and the type of tissue being tested. Nevertheless, the obtained results indicate that both PPARδ and PPARγ may be involved in the luteolytic pathways mediated by PGF_2α_ in the bovine CL.

Furthermore, we demonstrated that the inhibition of individual PPAR isoforms together with the FP receptor, and the simultaneous blockade of all PPAR isoforms without the parallel inhibition of the FP receptor, decreased *PTGS2* mRNA expression in the bovine CL explants during PGF_2α_-induced mechanisms related to the CL regression in vitro. This may also suggest the involvement of specific PPAR isoforms in the activation of the inter- and intra-cellular mechanisms involved in PGF_2α_-stimulated PGF_2α_ production. On the other hand, the mRNA of *PTGS2* in the bovine CL explants stimulated only with PGF_2α_ was increased as compared to the untreated explants. Previously, it has been demonstrated that PGF_2α_ secretion within the bovine CL increases during PGF_2α_-induced luteolysis [54], and thus, PGF_2α_ secreted in the CL may play a role as an autonomous amplification of uterine PGF_2α_ during luteolysis [55]. This auto-amplification loop system for PGF_2α_ production may aid in the progression towards CL luteolysis. Enzymes such as PTGS2 and PTGFS are known to participate in PGF_2α_ synthesis [56,57]. Shirasuna et al. [56] confirmed that the mRNA expression of key enzymes of PGF_2α_ biosynthesis was increased in the bovine CL after PGF_2α_ treatment. Moreover, in the study of Kumagai et al. [57], PTGS2 and PTGFS abundance significantly increased in cultured bovine luteal cells after 24 h of treatment with PGF_2α_, suggesting that the auto-amplification system of PGF_2α_ is mediated by PTGS2 and PTGFS. The obtained results are in accordance with previous findings [56,57] and confirm the effectiveness of the in vitro model applied in our study. However, further detailed studies regarding a direct interaction between PGs and PPARs in the bovine CL in connection with luteolytic signaling pathways are needed.

In the present study, the luteolytic effect of PGF_2α_ was also confirmed by the reduction in P_4_ secretion in the bovine CL explants following stimulation only with PGF_2α_ and/or preceding FP receptor blockade. The inhibition of P_4_ concentration after luteolytic PGF_2α_ treatment was shown previously by Pate and Condon [34]. Furthermore, Korzekwa et al. [58] confirmed that PGF_2α_ treatment decreased P_4_ secretion in the cocultures of all types of bovine CL cells. Moreover, in accordance with a previous report of Hryciuk et al. [59], the PGF_2α_ treatment of bovine CL explants in our study did not induce any significant changes in the mRNA expression of *StAR*, *P450scc* and *HSD3B1*, which are key enzymes mediating changes in P_4_ production during the estrous cycle [60].

The results of the present study suggest that the effect of PPAR on P_4_ release during PGF_2α_-induced luteolysis in vitro may be related to the regulation of the action of steroidogenic enzymes. Interestingly, in our study, P_4_ secretion decreased, and the mRNA expression of *HSD3B1* was also downregulated in the bovine PGF_2α_-treated CL explants where PPARγ was not blocked. Moreover, in the CL explants under the influence of PGF_2α_ in combination with PPARδ, the mRNA expression of *HSD3B1* also decreased. We can, therefore, assume that PPARγ and PPARδ may be potentially involved in P_4_ production through the regulation of steroidogenesis and in PGF_2α_-induced bovine CL regression. In contrast, PPARα seems to have limited involvement in those processes. However, further research is advisable.

Furthermore, the functional and structural changes observed in PGF_2α_-induced luteolysis depend on the autocrine and paracrine factors produced within the CL [61]. The decrease in P_4_ secretion occurs before the biochemical signs of structural luteolysis are observed, and the size of the CL is finally decreased [15]. Cytokines, including TNFα, which acts specifically through various receptors, TNFRSF1A (death receptors) and TNFRSF1B (survival receptors) [29,62] or NO [25,55], are known to act as mediators/modulators of PGF_2α_ luteolytic activity. Moreover, inducible NO isoforms contain endothelial nitric oxide synthase (eNOS) and iNOS enzymes responsible for NO synthesis in the bovine CL [29]. Previously, it was shown that PGF_2α_ increases NO in luteal cell culture [60]. In our study, we observed an increase in *TNFα* and its receptor, *TNFRSF1A*, and *iNOS* mRNA expression in the bovine CL explants after PGF_2α_ treatment, which confirms their participation in in vitro-induced CL regression.

Data describing how PPAR affects the mediators of luteolytic PGF_2α_ activity in the bovine CL are still lacking. Regarding PPAR, it was only reported that treatment with PPARγ agonist downregulated iNOS expression in ovarian macrophages [63]. In addition, the secretion of proinflammatory cytokines such as TNFα and interleukin (IL)-6 was inhibited after stimulation with PPARγ agonist in human granulosa-lutein cells [64]. It is difficult to relate these observations to the results of our research. In the present study, the mRNA expression of *TNFα*, *TNFRSF1B* and *iNOS* increased in the bovine PGF_2α_-treated CL explants where PPARγ was not blocked. Therefore, taking into account the obtained results and general information on the mechanisms of PGF_2α_-induced luteolysis in cows, we can assume that PPARγ could be a factor involved in the regulation of processes related to functional luteolysis in the bovine CL directly induced by PGF_2α_ and may not be involved in the regulation of other mediators of PGF_2α_ action, such as NO and proinflammatory cytokines. However, further investigation is warranted to study this hypothesis.

## 5. Conclusions

Molecular mechanisms of PPARα and PPARδ action in the luteolytic pathways are still not fully understood. However, this study provides novel information on PPARα, PPARδ and PPARγ in the CL in cattle. The obtained results indicate that the mRNA and protein expression of PPARs changes in the bovine CL throughout the estrous cycle and under the influence of PGF_2α_. We suggest that PPARγ, among all of the examined PPAR isoforms, seems to be a factor involved in the regulation of PGF_2α_-induced processes related to functional luteolysis in the bovine CL. It seems that in the bovine CL, PPARs may affect its regression at multiple sites. Further studies are needed to understand the role of PPAR in the PGF_2α_-induced processes related to functional luteolysis in the bovine CL and how its varying expression is regulated during the lifespan of the CL. Our study provides new perspectives for understanding the role of PPARs in cattle reproduction.

These findings help to expand the knowledge of the mechanisms of luteal regression in the bovine CL. In the long-term perspective, this could have practical application in the development of assisted reproductive techniques in domestic animals using an injection of exogenous PGF_2α_.

## Figures and Tables

**Table 1:** Oligonucleotide sequences used for quantitative PCR.

Gene Name	Primer Sequence (5′–3′)	Amplicon Length (bp)	Accession Number (GenBank)
*GAPDH*	F: CACCCTCAAGATTGTCAGCA	103	BC102589
	R: GGTCATAAGTCCCTCCACGA		-
*ACTB*	F: CCAAGGCCAACCGTGAGAAAAT	256	K00622
	R: CCACATTCCGTGAGGATCTTCA		
*RN18S1*	F: AAGTCTTTGGGTTCCGGG	365	AF176811
	R: GGACATCTAAGGGCATCACA		
*PPARα (PPARA)*	F: GTGGAGAGTTTGGCAGAACCAGA	163	NM_001034036.1
	R: TCCCACTGCCCAGCTCCGATC		
*PPARδ (PPARD)*	F: TCCGAAAGCCCTTCAGTGA	124	NM_001083636.1
	R: GGATGGCCTCCACCTGAGACA		
*PPARγ (PPARG)*	F: AGGACATTCCGTTCCCAAG	101	NM_181024.2
	R: GCCATGAGGGAGTTGGAAG		
*StAR*	F: GGTGGTGGCACGTTTTCAAT	79	Y17259.1
	R: CCTTGTCCGCATTCTCTTGG		
*P450scc*	F: CAGCATATCGGTGACGTGGA	139	K02130.1
	R: GGCCACCAGAACCATGAAAA		
*HSD3B1*	F: CTAATGGGTGGGGCTCTGAAA	473	NM_174343
	R: CACGCTGTTGGAAAGAGTCA		
*PTGS2*	F: TGTTTGCATTCTTTGCCCAG	158	NM_174445
	R: CATCCTTGAAAAGGCGCAG		
*PTGFS*	F: TGTGGTGCACGTATCACGACA	160	S54973
	R: AATCACGTTGCCGTCCTCATC		
*TNFα*	F: ATGAGGTAAAGCCCGTCAGC	132	NM_173966.3
	R: CCACGTTGTAGCCGACATCA		
*TNFRSF1A*	F: CTGGTGATTGTCTTCGGGCT	104	NM_174674.2
	R: TGCCCGCAAATGATGGAGTA		
*TNFRSF1B*	F: GTAGCTCAGAGGCGTCTTCC	74	NM_001040490.2
	R: GCCGCTGCAAACATTGACA		
*iNOS*	F: AAGCCGTGTTCTTCGCCTCG	135	DQ676956.1
	R: AGGCACAGCTGAACAAAGCCCC		

## Data Availability

None of the data were deposited in an official repository.

## Supplementary Materials

The following supporting information can be downloaded at: https://www.mdpi.com/article/10.3390/ani12121542/s1, Figure S1: The effect of inhibition of PPARα, PPARδ, PPARγ and PGF_2α_ receptor (FP) in the bovine PGF_2α_-treated corpus luteum (CL) explants on progesterone (P_4_) secretion on days 15–17 of the estrous cycle. The results are presented as a % of control. Presented results are the mean ± SEM from 9 animals. The asterisks indicate statistical differences in the experimental groups versus the control group (* *p* < 0.05) as determined by nonparametric one-way ANOVA Kruskal–Wallis followed by Dunn’s multiple comparisons test. The groups are marked as follows: C—control group (untreated CL explants), P—CL explants stimulated with PGF_2α_ (10^−6^ M); PAL—CL explants stimulated with FP antagonist (10^−5^ M) and PGF_2α_ (10^−6^ M); APAL 1/2—CL explants stimulated with FP antagonist (10^−5^ M), PPARα antagonist (10^−5^ M), PPARδ antagonist (10^−5^ M), and PGF_2α_ (10^−6^ M); APAL 2/3—CL explants stimulated with FP antagonist (10^−5^ M), PPARδ antagonist (10^−5^ M), PPARγ antagonist (10^−5^ M), and PGF_2α_ (10^−6^ M); APAL 1/3—CL explants stimulated with FP antagonist (10^−5^ M), PPARα antagonist (10^−5^ M), PPARγ antagonist (10^−5^ M), and PGF_2α_ (10^−6^ M); AP 1/2/3—CL explants stimulated with PPARα antagonist (10^−5^ M), PPARδ antagonist (10^−5^ M), PPARγ antagonist (10^−5^ M), and PGF_2α_ (10^−6^ M); Figure S2: The effect of inhibition of PPARα, PPARδ, PPARγ and PGF_2α_ receptor (FP) in the bovine PGF_2α_-treated CL explants on mRNA expression of StAR (A) P450scc (B) and HSD3B1 (C) on days 15–17 of the estrous cycle. The results are presented as a fold change. Presented results are the mean ± SEM from 9 animals. The asterisks indicate statistical differences in the experimental groups versus the control group (* *p* < 0.05; ** *p* < 0.01) as determined by nonparametric one-way ANOVA Kruskal–Wallis followed by Dunn’s multiple comparisons test. The groups are marked as follows: C—control group (untreated CL explants), P—CL explants stimulated with PGF_2α_ (10^−6^ M); PAL—CL explants stimulated with FP antagonist (10^−5^ M) and PGF_2α_ (10^−6^ M); APAL 1/2—CL explants stimulated with FP antagonist (10^−5^ M), PPARα antagonist (10^−5^ M), PPARδ antagonist (10^−5^ M), and PGF_2α_ (10^−6^ M); APAL 2/3—CL explants stimulated with FP antagonist (10^−5^ M), PPARδ antagonist (10^−5^ M), PPARγ antagonist (10^−5^ M), and PGF_2α_ (10^−6^ M); APAL 1/3—CL explants stimulated with FP antagonist (10^−5^ M), PPARα antagonist (10^−5^ M), PPARγ antagonist (10^−5^ M), and PGF_2α_ (10^−6^ M); AP 1/2/3—CL explants stimulated with PPARα antagonist (10^−5^ M), PPARδ antagonist (10^−5^ M), PPARγ antagonist (10^−5^ M), and PGF_2α_ (10^−6^ M); Figure S3: The effect of inhibition of PPARα, PPARδ, PPARγ and PGF_2α_ receptor (FP) in the bovine PGF_2α_-treated CL explants on mRNA expression of PTGS2 (A) PTGFS (B) TNFα (C) TNFRSF1A (D) TNFRSF1B (E) and iNOS (F) on days 15–17 of the estrous cycle. The results are presented as a fold change. Presented results are the mean ± SEM from 9 animals. The asterisks indicate statistical differences in the experimental groups versus control group (* *p* < 0.05; ** *p* < 0.01; *** *p* < 0.001) as determined by nonparametric one-way ANOVA Kruskal–Wallis followed by Dunn’s multiple comparisons test. The groups are marked as follows: C—control group (untreated CL explants), P—CL explants stimulated with PGF_2α_ (10^−6^ M); PAL—CL explants stimulated with FP antagonist (10^−5^ M) and PGF_2α_ (10^−6^ M); APAL 1/2—CL explants stimulated with FP antagonist (10^−5^ M), PPARα antagonist (10^−5^ M), PPARδ antagonist (10^−5^ M), and PGF_2α_ (10^−6^ M); APAL 2/3—CL explants stimulated with FP antagonist (10^−5^ M), PPARδ antagonist (10^−5^ M), PPARγ antagonist (10^−5^ M), and PGF_2α_ (10^−6^ M); APAL 1/3—CL explants stimulated with FP antagonist (10^−5^ M), PPARα antagonist (10^−5^ M), PPARγ antagonist (10^−5^ M), and PGF_2α_ (10^−6^ M); AP 1/2/3—CL explants stimulated with PPARα antagonist (10^−5^ M), PPARδ antagonist (10^−5^ M), PPARγ antagonist (10^−5^ M), and PGF_2α_ (10^−6^ M).
