# Supplementary material for: The Role of Peroxisome Proliferator-Activated Receptors in PGF2α-Induced Luteolysis in the Bovine Corpus Luteum
Source: Animals (Basel). 2022 Jun 14;12(12):1542. doi: 10.3390/ani12121542 (PMC9219485; doi:10.3390/ani12121542)
Supplement: Supplementary file 1 [file animals-12-01542-s001.zip › Supplementary Figure S3.pdf]

Supplementary Figure S3

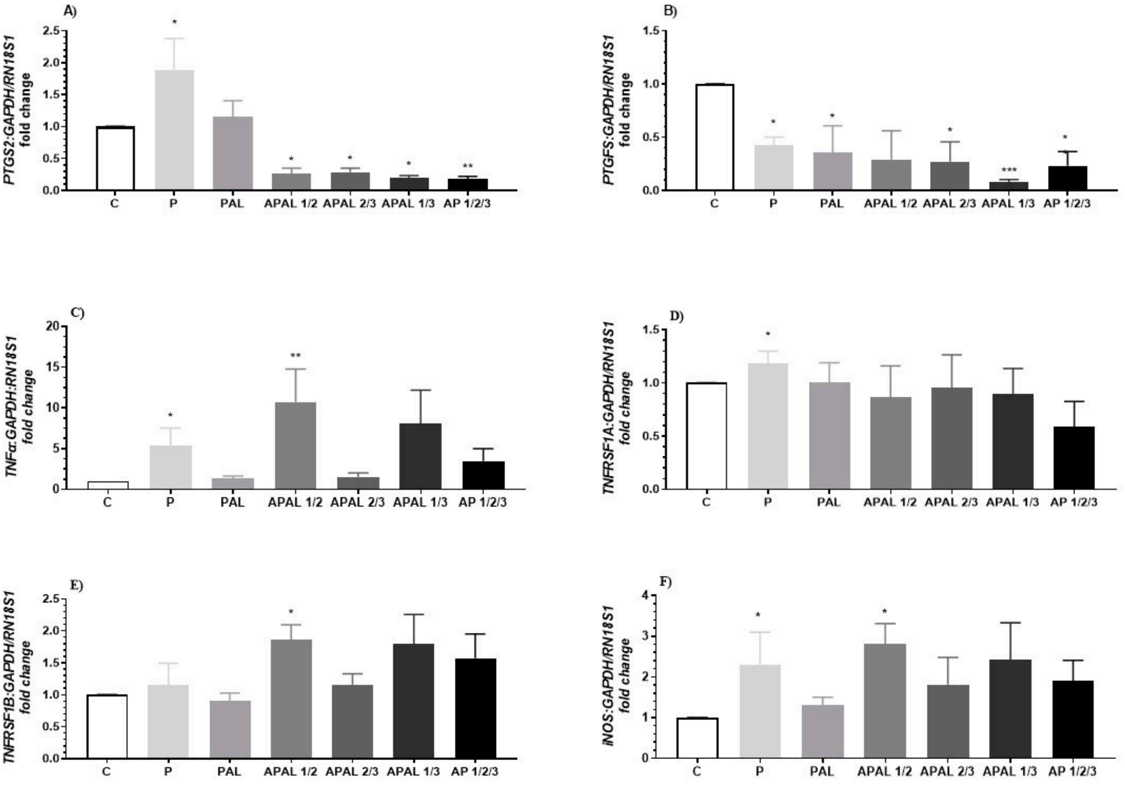

Supplementary Figure S3. The effect of inhibition of PPARα, PPARδ, PPARγ and PGF$_{2\alpha}$ receptor (FP) in the bovine PGF$_{2\alpha}$-treated CL explants on mRNA expression of PTGS2 (A) PGFS (B) TNFα (C) TNFRSF1A (D) TNFRSF1B (E) and iNOS (F) on days 15-17 of the estrous cycle. The results are presented as a fold change. Presented results are the mean ± SEM from 9 animals. The asterisks indicate statistical differences in the experimental groups versus control group (* $p < 0.05$; ** $p < 0.01$; *** $p < 0.001$) as determined by nonparametric one-way ANOVA Kruskal–Wallis followed by Dunn's multiple comparisons test. The groups are marked as follows: C–control group (untreated CL explants), P–CL explants stimulated with PGF$_{2\alpha}$ (10^{-6} M); PAL–CL explants stimulated with FP antagonist (10^{-5} M) and PGF$_{2\alpha}$ (10^{-6} M); APAL 1/2–CL explants stimulated with FP antagonist (10^{-5} M), PPARα antagonist (10^{-5} M), PPARδ antagonist (10^{-5} M), and PGF$_{2\alpha}$ (10^{-6} M); APAL 2/3–CL explants stimulated with FP antagonist (10^{-5} M), PPARδ antagonist (10^{-5} M), PPARγ antagonist (10^{-5} M), and PGF$_{2\alpha}$ (10^{-6} M); APAL 1/3–CL explants stimulated with FP antagonist (10^{-5} M), PPARα antagonist (10^{-5} M), PPARγ antagonist (10^{-5} M), and PGF$_{2\alpha}$ (10^{-6} M); AP 1/2/3–CL explants stimulated with PPARα antagonist (10^{-5} M), PPARδ antagonist (10^{-5} M), PPARγ antagonist (10^{-5} M), and PGF$_{2\alpha}$ (10^{-6} M).
